# The Effects of Palmar Cooling on Repeated Sprinting Ability: A Randomized Controlled Clinical Trial

**DOI:** 10.3390/s25061830

**Published:** 2025-03-15

**Authors:** Michael Brown, Jacob Daniels, Marli Crabtree, Kenneth Thompson, Joshua Murphy, William Pannell, Ryan McGlawn

**Affiliations:** 1 Department of Physical Therapy, School of Health Related Professions, University of Mississippi Medical Center, Jackson, MS 39216, USA; jbdaniels@umc.edu (J.D.); jmurphy1@umc.edu (J.M.); wpannell@umc.edu (W.P.); rmcglawn@umc.edu (R.M.); 2 Department of Anatomy, Duquesne University College of Osteopathic Medicine, Pittsburgh, PA 15219, USA; crabtreem1@duq.edu; 3 Department of Advanced Biomedical Education, School of Medicine, University of Mississippi Medical Center, Jackson, MS 39216, USA; klthompson2@umc.edu

**Keywords:** athletic performance, muscle fatigue, recovery, thermoregulation

## Abstract

**Highlights:**

**What are the main findings?**

- Palmar cooling potentially positively impacts repeated sprinting performance.
- Palmar cooling potentially reduces post-exercise muscle soreness.

**What are the implications of the main findings?**

- Using palmar cooling devices could augment activities that involve sprinting.
- Using palmar cooling could augment recovery following exercise.

**Abstract:**

Evidence supports the role of palmar cooling to improve exercise performance, especially with endurance and resistance activities. The aim of this randomized placebo-controlled trial was to explore the effects of palmar cooling on repeated sprinting performance and recovery. Fifteen graduate students were randomly assigned to either a palmar cooling intervention or placebo group (males: n = 8, females: n = 7; Avg. age: 24.06 yrs.) After a ten-minute warm-up, participants completed ten sixty-meter sprints that included two 180-degree changes of direction. Three bouts of two-minute intervention or placebo occurred during the study. Data for sprint times, heart rate, and RPE were collected throughout testing. A muscle soreness rating was collected via survey 48 h post intervention. Statistically and practically significant differences were found between groups for average sprint times, heart rate, and delayed onset muscle soreness. The intervention group utilizing palmar cooling demonstrated less degradation in sprint times, lower heart rate upon completion, and a lower soreness rate 48 h after testing. More research is needed with a larger sample size to determine if practical and statistically significant differences will be maintained and would allow for a more robust multivariant analysis, resulting in the findings being more generalizable to a larger population.

## 1. Introduction

The ability to perform repeated, short bouts of maximal to near-maximal sprinting is critical for various sport-related activities. In team sports activities, such as basketball, rugby, or soccer, the ability of a player to obtain possession of the ball is a matter of fractions of a second. Championships are won or lost because of tenths of a second. It has been estimated that a ~0.8% difference in sprint speed can determine which player gains possession in a 20 m sprint to the ball [1]. Neuromuscular fatigue associated with sprinting occurs rapidly and is a multifactorial physiological response [2]. Regardless of the physiological cause, skeletal muscle fatigue is directly associated with reduced sprinting ability.

Temperature plays a critical role in many human biological processes, with multiple studies demonstrating an optimal temperature range for muscle function [3,4,5]. Furthermore, energy production during exercise can create a significant amount of excess heat locally at the muscle and systemically throughout the body [3]. Additionally, a study suggests that thermoregulatory patterns and their physiologic mechanisms may be different between the muscle and tendon [6]. To regulate heat from metabolic processes and the external environment, the body uses radiation, conduction, and evaporation to continuously work to maintain a steady normal range or “set point” [7]. As core body temperature rises, the body’s mechanisms attempt to shift to create more significant heat loss than production, as able. Peak exercise intensity can raise the core body temperature to a febrile-like state that results in a systematic feeling of being overheated. This process works to evoke a behavioral modification that causes a shift in activity towards reducing the temperature, suggesting that the system regulates temperature in anticipation or avoidance of a potentially catastrophic event [8]. This means that high-intensity exercise can generate a rise in core body temperature that signals the body to reduce heat production, potentially by limiting muscle function through downregulation of the central nervous system signal to skeletal muscle. ATP production could also be restricted due to this shift towards heat loss resulting in reduced muscular output.

Palmar cooling devices attempt to leverage the conductive component of our system’s cooling mechanism through two main strategies. First, palmar cooling capitalizes on the anatomical structure of glabrous skin. Glabrous skin is found on the soles of the feet, the palms of the hands, and the hairless portions of the face. This skin effectively transfers heat from the body’s core through a unique vascular component called arteriovenous anastomoses, where arterioles and venules join. Arteriovenous anastomoses allow for blood to bypass the capillary network and affect a greater volume of blood with decreased pressure due to the size of venules and arterioles. Therefore, glabrous skin potentially serves as an integral heat radiator during bouts of elevated temperature [9] and may hasten heat loss compared to other physiological strategies [10]. Second, palmar cooling leverages the heat transfer properties of continuous-flow water. Water removes heat through conduction at a far higher rate than air, and the continuous flow eliminates the development of a thermal barrier between the surfaces of the palm and the cooling device. Palmar cooling offers a potential solution to reduce thermoregulatory strain during high-intensity activity, where short bouts of rest are provided, and therefore improve performance [11].

Previous research has demonstrated the effectiveness of modulating thermoregulation through cooling the glabrous regions [12] and an increase in work volume for both strength training and aerobic treadmill activities for individuals utilizing palmar cooling for heat extraction [3,13]. Palmar cooling has also been shown to reduce fatigue and increase rowing work volume [14]. Another study described palmar cooling’s positive impact on heart rate during strength, power, and endurance exercise testing and suggested future research should consider higher-intensity exercise activities such as repeated sprinting [15]. The role of palmar cooling in repeat sprinting remains unexplored. Sprinting is a unique physiological challenge, requiring rapid ATP resynthesis and neuromuscular coordination, both of which are susceptible to heat-related fatigue [2]. Unlike endurance activities, where sustained cooling strategies have been shown to enhance performance [13], it is unclear whether palmar cooling can effectively mitigate performance decline in short, high-intensity sprinting bouts, especially with maximal effort and changes of direction. Furthermore, past studies on palmar cooling have focused primarily on aerobic exercise [11,12,13,14] and resistance training [3,10]. However, none have specifically examined its effects on repeated sprinting ability, a key determinant of success in many competitive sports. Given the rapid onset of neuromuscular fatigue during repeated sprinting and the potential for heat accumulation to accelerate performance decline, identifying effective cooling strategies is of both scientific and practical importance. This study is the first to assess whether palmar cooling can enhance repeated sprinting ability by delaying fatigue and improving recovery between sprints. By addressing this gap, the findings could provide valuable insights for athletes, coaches, and sports scientists seeking to optimize performance through targeted thermoregulation strategies. The purpose of this randomized placebo-controlled trial was to explore the effects of palmar cooling on repeated sprinting performance and recovery.

## 2. Materials and Methods

### 2.1. Participants

UMMC students within the School of Health Related Professions were recruited to participate in this study in June 2024. During the recruitment process, students were informed that their participation was completely voluntary and their decision did not impact their standing as a student at UMMC. The research investigator that recruited participants did not recruit participants for whom he served as an instructor of record during the recruitment and testing time frame. A recruitment email was sent to students at the School of Health Related Professions and recruitment occurred through face-to-face communication with students. When individuals expressed interest via email in participating in the study, a UMMC study investigator educated them on the study and scheduled a testing time. During the face-to-face recruiting, participants were educated on the study and those who were interested signed up via a hard copy using their email addresses. A reminder email was sent to participants reminding them of the testing time that they signed up for. Participants were not enrolled until informed consent was obtained. Fifteen healthy School of Health Relations Professions graduate students (8 male, 7 female) in their twenties at the University of Mississippi Medical Center (UMMC) participated in this randomized placebo-controlled trial. Graduate students were included due to convenience, general well-being, and relatively active lifestyle. The following exclusion criteria were applied for subject participation: (1) Self-reported injury, illness, or diagnosis; (2) Explicit instructions from a healthcare provider to avoid exercising at maximal capacity; (3) Previous use of a palmar cooling device. Participant characteristics are provided in Table 1. All participants provided written informed consent prior to testing, and the study was approved by the UMMC institutional review board (UMMC-IRB-2023-317). This study was also registered with ClinicalTrials.gov (NCT06356142).

### 2.2. Experimental Design

The study was completed in one day on an indoor gym basketball court that is cooled and climate-controlled at a steady-state temperature. All participants were fitted with a Polar H10 chest strap heart monitor that gathered heart rate data continuously throughout the experiment. The Polar H10 was used due to its demonstrated reliability [16,17]. Participants were blinded to group assignment and randomized into two groups (Group A: Intervention and Group B: Placebo) by drawing randomly generated study identification numbers from the lead investigator. Three-digit numbers represented the control group, and four digits represented the intervention group. The intervention group received a cooling intervention during three specified rest periods that works by supplying a pumped, continuous flow of cool water to the glabrous skin of the palm of the hand in a mitt-like design. The placebo-controlled component allowed the participants to place a hand in the mitt, but a device failsafe was manipulated to prevent the delivery of cool water to the palm. Therefore, the device could be turned on and used without pumping water through the bladder of the mitt, ensuring no active cooling occurred. All participants received the same description related to cooling, that due to the warming from sprinting, the bladder should feel cool but not cold and may be imperceptible. Due to the participants being blinded to group assignment, this deception created an environment where all participants believed that they were receiving the intervention. Participants completed a warm-up, a two-minute rest interval, five sixty-meter sprints that included two 180-degree changes of direction, a two-minute rest interval, and another bout of five sixty-meter sprints that included two 180-degree changes of direction. The participants had twenty seconds to return to the starting point between sprints. Research assistants collected time data using handheld stopwatches at the finish point of the sprints. A follow-up survey on muscle soreness was emailed forty-eight hours after study participation completion. Data points were collected for sprint times, heart rate, and rating of perceived exertion (RPE) throughout the study and are outlined in Table 2. Muscle soreness rating was collected via a survey that the participants completed forty-eight hours after study participation. The data collectors were blinded to group assignment except for the unblinding of the collector for 1 participant due to a scheduling conflict. However, the data do not suggest any significant influence on the information obtained for this participant.

### 2.3. Experimental Protocol

Testing consisted of four participants at a time on Friday, 28 June 2024 between 9 a.m. and 2 p.m. CST. Four testing sprint lanes (two aligned with the intervention device and two aligned with the placebo device) were used with tape markers twenty meters apart. Two study personnel were placed in each lane. One researcher was responsible for recording data (heart rate, RPE, and sprint times), while the other kept time. During each time slot, the participants completed a structured warm-up led by an investigator for ten minutes, which included jogging and plyometrics activities. Between sprints, participants were not cued to go on demand. Instead, participants were given instructions when roughly 15 s had passed that they had 5 s remaining and should sprint when they were ready. Similar instructions were given following each two-minute rest interval. Participants sprinted with maximal effort from the starting point (A) to the twenty-meter point (B) turned 180 degrees, returned to A, turned 180 degrees, and finished the sprint by returning to B. The total distance for the sprint was sixty meters with two 180-degree changes of direction. Participants completed five sprints that included the window of twenty seconds allotted to return to the starting point for each sprint. After five sprints, each participant received a two-minute rest interval and received either the intervention or placebo based on the original group assignment. Immediately following the rest interval, the participants completed the same five sprint sequence, ending in a total of ten sprints. After the last sprint, participants received either the intervention or the placebo based on the original group assignment. Participants were allowed to leave at this time. Forty-eight hours later, participants received an emailed survey and rated their muscle soreness.

### 2.4. Ethical Considerations

All participants provided written informed consent prior to testing, and the study was approved by the UMMC institutional review board (UMMC-IRB-2023-317). This study was also registered with ClinicalTrials.gov (NCT06356142).

### 2.5. Statistical Analysis

Participants in this study were randomly assigned to control and experimental groups. Beyond randomization and graphic/visual analysis, sensitivity analysis and propensity score matching were also utilized to address any possible confounding variables. Each of the outcomes was measured as continuous variables and analyzed using parametric statistical tests. Prior to analysis, the data were analyzed graphically and visually to aid in identifying confounding variables to ensure data quality and to determine whether the required assumptions for statistical analysis were met. Because the continuous outcomes were considered between two discrete and independent groups, analysis of variance (ANOVA) was used to examine the relationship between each of the outcomes (the time to complete each sprint, continuous heart rate monitoring, RPE measured during the rest intervals, and a muscle soreness rating two days after the training event) and each of the groups (palmar cooling intervention and placebo palmar cooling intervention).

Additionally, independent samples *t*-tests were used to analyze the relationship between the average differences in sprint times between the control and experimental groups. Furthermore, the differences in sprints one and six, sprints one and ten, heart rates five and four, heart rates six and four, the RPEs four and three, RPEs six and two, heart rates seven, and soreness ratings between the control and intervention groups were analyzed using independent samples *t*-tests. The alpha value for all statistical analyses was set to 0.05. In addition to determining statistical significance, power as well as Cohen’s d or partial eta-squared effect sizes were calculated, analyzed, and reported for all relationships analyzed using ANOVA or independent samples *t*-tests. Recommended parameters were used to evaluate the size of the Cohen’s d effect, with a small effect ranging from 0.2 to 0.49, a medium effect ranging from 0.5 to 0.79, and a large effect from 0.8 and above [18]. For results that utilized partial eta-squared, recommended parameters were also used, with a small effect ranging from 0.01 to 0.059, a medium effect ranging from 0.06 to 0.139, and a large effect from 0.14 and above [18]. All *p*-values were reported to the thousandth place, while all other numerical results were rounded to the hundredth.

## 3. Results

The study sample consisted of 15 total participants. Within this study, participants were randomly assigned to either an intervention or control group and blinded to group assignment. Ultimately, nine participants were assigned to the intervention group and six were assigned to the control group. For each relationship statistically analyzed, the total number of participants used in the analysis varied, as the researchers excluded participants based on the specific data being observed. The data point exclusion was connected to the withdrawal of two participants at around half completion of the activity, one timing error reported by a researcher, and one participant not completing the survey. The data points generated by each participant were used if collected and not used when unavailable. The total number of participants, the number of participants in the intervention and control groups, and the reasons why these exclusions were made will be stated with each statistical analysis.

Responses to the demographic portion of the questionnaire are presented in Table 1. Age was reported by participants using their birth date, including day, month, and year. Participants ranged in age from 22 to 27 years old at the time of the study.

Using an independent samples t-test, the difference in the seventh recorded heart rate, the first recorded heart rate after the second rest interval, between the control and intervention groups was statistically significant (t (11) = 2.276, *p* = 0.044). The average heart rate for the intervention group was nearly 14 beats lower than the average for the control group. One participant in the intervention group was excluded from this analysis because they became ill before the second set of sprints, and one participant in the control group was excluded for the same reason. These exclusions resulted in five participants in the control group and eight in the intervention group. Using Cohen’s d, a large effect size was found. The average heart rates for each group are presented in Table 3, along with the *p*-value and effect size.

The soreness ratings between the control and intervention groups were found to be statistically different (t (12) = 2.190, *p* = 0.049). The intervention group averaged a 1.83 lower soreness rating when compared to the control group. For this analysis, there were six participants in the control group and eight in the intervention, as one participant in the intervention group did not complete the follow-up questionnaire. A large effect size was found using Cohen’s d. All statistical results and the average soreness ratings for each group are presented in Table 3.

The differences between times one and six and times one and ten did not significantly vary between the control and intervention groups. Although statistical significance was not found, both had significant effect sizes. The difference in times one and six between the control and intervention groups had a large effect size, as measured by Cohen’s d. Using Cohen’s d, a medium effect size was observed when analyzing the differences between times one and ten recorded with the control and intervention groups. The average differences for each group between each of these times and the statistical results are presented in Table 3.

The differences between heart rates five and four and heart rates six and four for the control and intervention groups were analyzed using independent samples *t*-tests and were found to be statistically insignificant. All fifteen participants were used in these analyses. Using Cohen’s d, small effect sizes were observed for both relationships. All statistical results and average differences are presented in Table 3.

The difference between the fourth and third recorded RPEs for the control and intervention groups were statistically analyzed using independent samples *t*-tests, and no significance was found. There were no participant exclusions for this analysis. Cohen’s d determined no significant effect size for this relationship. Contrastingly, when analyzing the differences between the sixth and second recorded RPEs for the control and intervention groups, one participant in the control and one participant in the intervention group were excluded due to becoming ill before the second set of sprints. This analysis also showed no statistical significance, but using Cohen’s d, a medium effect size was observed. All related average differences for the RPEs and the statistical results are presented below in Table 3.

An analysis of variance (ANOVA) was the statistical method utilized for the remainder of the analyses discussed.

Statistically significant differences were found between biological sex and the seventh recorded heart rate between the control and intervention groups (F (3, 12) = 4.189, *p* = 0.041). Partial eta-squared found a large effect size. As discussed previously, two participants were excluded from the analysis involving the seventh recorded heart rate due to becoming ill, one being in the control and the other in the intervention group. The differences in the seventh recorded heart rate in males and females in the control and intervention groups, as well as the statistical results, are presented below in Table 4.

The soreness rating, the differences between times one and six, and the differences in RPEs four and three between males and females in the control and intervention groups were not significantly different. Using partial eta-squared, a large effect size was found for each relationship. The average soreness ratings, differences in times one and six, differences in RPEs four and three, the number of participants used for each analysis, and the associated statistical results are listed in Table 4.

There were no statistically significant differences between males and females in the control and intervention groups when analyzing the differences between times one and ten, heart rates five and four, heart rates six and four, and RPEs six and two. Partial eta-squared found varying effect sizes, and these are listed below in Table 4, along with *p*-values, the number of participants used in each analysis, and the average associated outcomes.

The following variables showed no statistical significance when analyzed in relation to activity levels between the control and intervention groups. For the following analyses, the participants were categorized into binary groups based on their activity levels, which categorized all participants who reported a weekly activity level below 150 min together and all participants who reported a weekly activity level equal at or above 150 min together. All listed relationships did show large effect sizes, which were calculated using partial eta-squared. All participant numbers, averages, and effect sizes and their interpretations are presented below in Table 5.

## 4. Discussion

To the authors’ knowledge, no prior study has been completed to explore the effects of palmar cooling on repeated sprint performance. This study assessed the effects of utilizing a palmar cooling device on sprint times, heart rate, RPE, and delayed onset muscle soreness (DOMS). These variables are key physiological indicators of work output, fatigue, and recovery rate. Heart rate and RPE are two commonly used methods of measuring exercise intensity and recovery from exercise. Sprint times are expected to increase with subsequent maximal sprinting and, therefore, provide insight into the individual’s ability to maintain work output and short-term recovery, while DOMS provides information into protracted recovery from strenuous activity. Exercise performance and/or cessation is most frequently attributed to metabolic [19], neuromuscular, cardiovascular, and respiratory factors. Typically, discussion centers on energy source depletion, by-product accumulation, nervous system fatigue, and/or oxygen deficiencies [20]. Less emphasis is placed on the thermoregulatory components and their influence on exercise performance outside of catastrophic events such as heat illness or heat stroke. Research describes a potential decrease in skin temperature for both muscle and tendon as the isolated muscle fatigues following a prescribed fatiguing protocol using electrical stimulation and volitional maximal isometric contractions [6]. While we did not collect skin or core body temperature measurements, we did attempt to manipulate temperature using the palmar cooling intervention. To determine the performance effect of palmar cooling, we measured the performance indicators as outlined.

### 4.1. Sprint Times

The ability to sustain sprint performance over long durations offers a competitive advantage in athletic competitions. Fatigue can dictate the outcome of such events, and its mitigation, in many cases, is the decisive factor between victory and defeat. The expectation for sprint times to generally slow or increase as the repetitions or workload increased was demonstrated in the present study. However, as hypothesized, the performance of the group receiving the palmar cooling intervention degraded much less. These findings align with increased running distance [11,12,13] and increased rowing distance achieved [14] for participants receiving palmar cooling while exercising and during intermittent rest periods suggesting that cooling provides increased performance and work volume. The intervention group slowed by an average of 1.74 s between time 1 and time 10, while the control group slowed by an average of 2.76 s during the same period. While this finding was not statistically significant, key moments in sports, as previously discussed, can be decided by less than a 1 percent difference in sprint speed. Our findings demonstrate a 2.76 percent difference between groups in average final sprint speeds. This slowing variance was also demonstrated in the difference between the intervention times, 1.08 s, and the control times, 1.80 s, when comparing the average of time 1 to the average of time 6. This comparison between the average of time 1 and time 6 is interesting because the sixth sprint occurred immediately following a two-minute intervention or placebo period. The intervention also seemed to have a large impact on females at both the average difference between time 1 and time 6 and the average difference between time 1 and time 10 being 0.87 and 1.55 s, respectively, for the intervention group and 1.72 and 3.12 s, respectively, for the control group. Our findings indicate significant differences in post-intervention sprint times between intervention and control groups, with a large effect size and significant difference. However, one study examined the effects of palmar cooling on vertical jump height, isometric knee extension peak force, and a timed step-up protocol with no significant findings on any performance indicators except for heart rate [15]. Another study reported no performance benefits for pull-ups, push-ups, or leg extension output [21]. For studies that demonstrated no impact, there is potential that the participants did not exercise to a level of high thermal strain where thermoregulatory interventions would provide the most benefit. Practically, non-invasive palmar cooling during short breaks in action (e.g., timeouts, between quarters, etc.) appears to enhance performance sustainability when compared to standard rest conditions. This study demonstrates that even a brief, two-minute rest period with palmar cooling leads to practically significant differences and medium to large effect sizes in performance relative to a control group with equivalent rest periods.

Potentially, the main factor driving these results is the nervous system’s effort to maintain a state of homeostasis through a temperature set point. As the body’s temperature rises with exercise, the nervous system shifts its state to work toward heat loss. In an overheating subject, the central nervous system monitors the rate of elevation in core body temperature and anticipates thermal catastrophe. In response, the brain downregulates efferent skeletal muscle activation [22], thereby reducing work output and slowing the rise in core body temperature [8]. Palmar cooling may influence this physiologic feed-forward mechanism by lowering internal body temperature and alleviating CNS-driven skeletal muscle inhibition. This mechanism is also described as the “central fatigue response” [23]. Therefore, it is hypothesized that an intervention group utilizing palmar cooling will sustain higher levels of skeletal muscle recruitment over multiple high-intensity bouts, thus mitigating fatigue-induced declines in sprint performance. Our findings observed that maintenance of sprint times aligns with the proposed physiological mechanism, warranting further investigation into the exact impact of palmar cooling on core temperature regulation.

### 4.2. Heart Rate

Heart rate is also commonly used as a performance metric for exercise activities. As exercise or workload increases, the heart rate typically rises accordingly to meet the oxygen demand needed to complete the activity. Furthermore, blood circulation plays a vital role in thermal regulation. In a state of desired heat loss, the circulatory system carries heated blood from the body’s core to the skin to allow for heat radiation and dissipation, which could also increase heart rate as the body needs to work harder to lower its core temperature. One study found that for every degree (Celsius) rise in temperature, heart rate increases by about 12.3 beats per minute [24]. Therefore, a more efficient system of thermal regulation would decrease the demand on the heart to pump more heated blood to the skin. As described earlier, our findings show that the heart rate immediately following the final sprint was significantly higher in the control group versus the intervention group. This finding was also demonstrated with rowing and exercise testing [14,15] and supports the potential of the palmar cooling device to diminish the cardiac workload by reducing the body’s demand on the circulatory system to dissipate heat.

### 4.3. RPE

RPE is a performance measure typically used to allow the participant to gauge their perception of activity difficulty. The present study used the original Borg scale for ratings that range from 6 to 20 with lower numbers signifying minimal to no exertion and higher numbers signifying near max to maximal exertion. Due to the impact of palmar cooling on sprint performance and heart rate, a positive impact was expected on the subjective perception of the repeated sprinting activity. However, no statistically significant differences were observed between groups for RPE. It is possible that the perceived intensity of the activity did not impact the demonstrated performance gains.

### 4.4. DOMS

DOMS typically occurs after strenuous exercise activities, including frequent or extended eccentric contractions. Related activities often include deceleration components such as changing directions while sprinting and running downhill. Other common components, such as micro-tearing, inflammation, metabolite accumulation, and nociceptor activation, may also contribute to DOMS [25,26]. Less discussion is attributed to the role of temperature and DOMS. One study found that a warm-up prior to eccentric exercise results in decreased DOMS [27]. There is potential that the increased metabolic activity related to energy demand might contribute to muscle damage. Our findings suggest that the palmar cooling intervention seems to reduce some factors related to DOMS.

### 4.5. Strengths and Limitations

Strengths of this study include the randomized, blinded design with data collection occurring on the same day in a temperature-controlled environment. The participants were close in age, and a relatively equal mix of male and female participants was distributed among the intervention and control groups. Furthermore, the use of a placebo for the control group reduces the impact of intervention perception on the results. The limitations of this include a small population size with varied self-reported activity fitness levels. Full data sets were not collected for all fifteen participants, but the analysis was only completed using captured data. Due to its novelty in sports performance, no specific palmar cooling device has been validated. The device used in this study has shown a positive impact on performance in non-peer-reviewed case studies. Additionally, sprint times were collected using handheld stopwatches, which introduces a greater potential for human error, but studies have shown that manual timing is a reliable approach [28,29]. Incidentally, more participants were randomly assigned to the intervention group as compared to the control which could potentially affect the comparison, especially with such a small sample size.

## 5. Conclusions

The results of this study suggest that palmar cooling is a viable, non-invasive strategy to enhance repeated sprint performance with minimal disruption to activity. This has direct implications for athletes and coaches in high-intensity, intermittent sports such as soccer, basketball, rugby, and American football, where fatigue accumulation can impair performance in crucial moments. By integrating palmar cooling during rest periods, athletes may experience improved sprinting capacity, reduced fatigue, and potentially faster recovery between efforts. Given that cooling interventions are typically resource-intensive or impractical during competition, this study provides an accessible, efficient method that can be seamlessly incorporated into training and competition strategies.

To strengthen these findings, future research should prioritize assessing the direct effects on core temperature using a larger, well-matched group of participants with similar training backgrounds for repeated sprinting activities. Future work should also explore how this intervention could be adapted for different competitive settings and environmental conditions. Additionally, investigating the long-term effects of integrating palmar cooling into training programs could provide deeper insights into its role in fatigue management and injury prevention. Examining variations in cooling duration, frequency, and environmental conditions would further refine best practices for its implementation. Future research could also attempt to quantify the change and rate of change for core body temperature with respect to prolonged exercise.

The social and scientific significance of this research extends beyond elite sports performance. Scientifically, this study contributes to the growing body of literature on thermoregulation and exercise physiology, specifically highlighting a novel application of palmar cooling in high-intensity, repeated sprint efforts. It also provides insight into non-invasive strategies for enhancing performance, which could have broader implications for sports science, rehabilitation, and occupational settings where heat stress is a concern.

## Figures and Tables

**Table 1:** Demographics.

Variables	Number
Mean Age	24.06 (1.31)
Number of Females	7
Number of Males	8
Intervention Group	9
Females	4
Males	5
Control Group	6
Females	3
Males	3
Average Weekly Exercise (min)	
1–59	3
60–99	2
100–149	3
150 or Greater	7

**Table 2:** Data descriptions.

Data	Description	Units Measured
HR 1	Participant heart rate taken immediately following the warm-up	Beats per minute
HR 2	Participant heart rate taken 1 min after warm-up	Beats per minute
HR 3	Participant heart rate taken 2 min after warm-up/just before sprint 1	Beats per minute
HR 4	Participant heart rate taken immediately after sprint 5	Beats per minute
HR 5	Participant heart rate taken 1 min after sprint 5	Beats per minute
HR 6	Participant heart rate taken 2 min after sprint 5/just before sprint 6	Beats per minute
HR 7	Participant heart rate taken immediately after sprint 10	Beats per minute
HR 8	Participant heart rate taken 1 min after sprint 10	Beats per minute
HR 9	Participant heart rate taken 2 min after sprint 10	Beats per minute
RPE 1	Participant RPE taken immediately following the warm-up	Borg Scale (6–20)
RPE 2	Participant RPE taken 2 min after warm-up/just before sprint 1	Borg Scale (6–20)
RPE 3	Participant RPE immediately after sprint 5	Borg Scale (6–20)
RPE 4	Participant RPE taken 2 min after warm-up/just before sprint 6	Borg Scale (6–20)
RPE 5	Participant RPE taken immediately after sprint 10	Borg Scale (6–20)
RPE 6	Participant RPE taken 2 min after sprint 10	Borg Scale (6–20)
Time 1	Participant time to complete sprint 1	Seconds
Time 2	Participant time to complete sprint 2	Seconds
Time 3	Participant time to complete sprint 3	Seconds
Time 4	Participant time to complete sprint 4	Seconds
Time 5	Participant time to complete sprint 5	Seconds
Time 6	Participant time to complete sprint 6	Seconds
Time 7	Participant time to complete sprint 7	Seconds
Time 8	Participant time to complete sprint 8	Seconds
Time 9	Participant time to complete sprint 9	Seconds
Time 10	Participant time to complete sprint 10	Seconds

**Table 3:** Variable averages between control and intervention groups.

Factor	n (I)	n (C)	I ± SD	C ± SD	Cohen’s d	Effect Size	*p*-Value
HR 7	8	5	175.13 ± 10.08	188.80 ± 11.30	1.30	Large	0.044
Soreness	8	6	2.00 ± 1.60	3.83 ± 1.47	1.20	Large	0.049
Difference between times 1 and 6 (s)	8	4	−1.08 ± 1.04	−1.80 ± 0.19	0.82	Large	0.096
Difference between times 1 and 10 (s)	8	4	−1.74 ± 2.46	−2.76 ± 1.18	0.76	Medium	0.458
Difference between HR 5 and HR 4 (bpm)	9	6	−34.56 ± 6.69	−37.00 ± 10.02	0.30	Small	0.578
Difference between HR 6 and HR 4 (bpm)	9	6	−46.33 ± 8.75	−50.17 ± 13.12	0.36	Small	0.506
Difference between RPE 4 and RPE 3	9	6	−4.22 ± 2.68	−4.17 ± 3.25	0.02	-	0.972
Difference between RPE 6 and RPE 2	8	5	4.50 ± 2.00	3.60 ± 2.07	0.44	Medium	0.453

**Table 4:** Analysis of impact of biological sex.

Factor	n (I)	n (C)	I ± SD	C ± SD	Partial Eta-Squared	Effect Size	*p*-Value
HR 7					0.58	Large	0.041
Male	4	2	168.25 ± 10.44	183.50 ± 12.02			
Female	4	3	182.00 ± 1.41	192.33 ± 11.68			
Soreness rating					0.32	Large	0.253
Male	5	3	2.00 ± 1.58	4.33 ± 1.53			
Female	3	3	2.00 ± 2.00	3.33 ± 1.53			
Times 1 and 6					0.19	Large	0.614
Male	4	2	−1.29 ± 1.17	−1.88 ± 0.04			
Female	4	2	−0.87 ± 1.02	−1.72 ± 0.30			
Times 1 and 10					0.07	Medium	0.888
Male	4	2	−1.93 ± 3.66	−2.40 ± 0.31			
Female	4	2	−1.55 ± 0.76	−3.12 ± 1.90			
HR 5 and HR 4					0.07	Medium	0.846
Male	5	3	−36.40 ± 4.34	−36.67 ± 14.98			
Female	4	3	−32.35 ± 9.03	−37.33 ± 5.13			
HR 6 and HR 4					0.10	Medium	0.762
Male	5	3	−49.20 ± 6.06	−50.67 ± 17.93			
Female	4	3	−42.75 ± 11.15	−49.67 ± 10.41			
RPE 4 and RPE 3					0.19	Large	0.501
Male	5	3	−4.40 ± 1.52	−2.33 ± 3.51			
Female	4	3	−4.00 ± 4.00	−6.00 ± 2.00			
RPE 6 and RPE 2					0.063	Medium	0.892
Male	4	2	4.50 ± 1.73	4.00 ± 2.83			
Female	4	3	4.50 ± 2.52	3.33 ± 2.08			

I, intervention; C, control.

**Table 5:** Analysis of impact of activity level.

Factor	n (I)	n (C)	I ± SD	C ± SD	Partial Eta-Squared	Effect Size	*p*-Value
HR 7					0.52	Large	0.077
Act lev 0	3	3	172.33 ± 15.89	182.33 ± 7.51			
Act lev 1	5	2	176.80 ± 6.50	198.50 ± 9.19			
Soreness rating					0.41	Large	0.141
Act lev 0	3	4	3.00 ± 1.00	3.75 ± 0.96			
Act lev 1	5	2	1.40 ± 1.67	4.00 ± 2.83			
Times 1 and 6					0.17	Large	0.656
Act lev 0	3	3	−1.28 ± 1.45	−1.78 ± 0.23			
Act lev 1	5	1	−0.96 ± 0.95	−1.85 ± 0.00			
Times 1 and 10					0.27	Large	0.439
Act lev 0	3	3	−3.20 ± 3.12	−2.95 ± 1.37			
Act lev 1	5	1	−0.87 ± 1.77	−2.18 ± 0.00			
HR 5 and HR 4					0.14	Large	0.633
Act lev 0	4	4	−35.75 ± 4.03	−34.25 ± 10.44			
Act lev 1	5	2	−33.60 ± 8.65	−42.50 ± 9.19			
HR 6 and HR 4					0.16	Large	0.583
Act lev 0	4	4	−44.50 ± 7.85	−46.50 ± 14.82			
Act lev 1	5	2	−47.80 ± 10.03	−57.50 ± 6.36			
RPE 4 and RPE 3					0.13	Large	0.673
Act lev 0	4	4	−5.50 ± 3.70	−3.75 ± 3.30			
Act lev 1	5	2	−3.20 ± 1.10	−5.00 ± 4.24			
RPE 6 and RPE 2					0.31	Large	0.324
Act lev 0	3	3	4.67 ± 1.15	2.33 ± 1.53			
Act lev 1	5	2	4.40 ± 2.51	5.50 ± 0.71			

I: intervention, C: control.

## Data Availability

Data related to this project are available from the researchers.

## Abbreviations

The following abbreviations are used in this manuscript:

MDPI	Multidisciplinary Digital Publishing Institute
DOAJ	Directory of Open Access Journals
UMMC	University of Mississippi Medical Center
SHRP	School of Health Related Professions
HR	heart rate
RPE	rating of perceived exertion
DOMS	delayed onset muscle soreness
